# Diphtheria; with Report of a Case Followed by Paralysis

**Published:** 1889-09

**Authors:** P. C. Coleman

**Affiliations:** Colorado, Texas


For Daniel's Texas Medical Journal.]

### DIPHTHERIA, WITH REPORT OF A CASE FOLLOWED BY PARALYSIS.

BY P. C. COLEMAN, M. D., COLORADO, TEXAS.

THERE is not a subject of medical literature of the present day of more importance than the one of diphtheria.

When we consider the fact that previous to 1860 diphtheria was comparatively a rare disease in this country, so rare that in the report of the board of health of Philadelphia in 1859 no cases of diphtheria were reported, and in New York in 1883 there were 2090 deaths out of 3500 cases, over 59 per cent.; and when we see the literature of the day from all parts of the country treating of the ravages of this disease with its fearful rate of mortality, may it not be denominated the modern scourge?

Does it seek its victims alone in the hut, where comfort and luxury never enter, or among the destitute, surrounded by the filth of the crowded city?

Ask the physicians who live in the rural districts, or the one in the city,—and they will tell you they see diphtheria where there is all in the way of modern hygienic surroundings to prevent disease of any kind, and still they have this disease in its most malignant form, where it would least be expected.

It is not, however. the intention of this paper to enter into any lengthy discussion of the pathology, symptoms, diagnosis, sequelæ, etc., of the disease, but to treat specially of the following case:

I saw S. S., aged four years, on December 23, at 11 p.m. The parents of the child gave the following history of the case: For several days previous they noticed slight cough, fever, at times very high, loss of appetite, and child complained of her throat hurting her on swallowing; on several occasions nausea and vomiting; bowels somewhat constipated. I found the following condition: Pulse 130, very weak; respiration rapid and irregular; the child every few minutes gasping for breath. Upon examination of throat the tonsils and posterior part of the pharynx were covered with a membraneous deposit almost as white as cotton, with abrupt, well defined margin. Submaxilary glands enlarged and tender. Child had refused nourishment for the past twenty-four hours, and when forced to swallow, did so with great difficulty.

This child had lived for the past year in this rare atmosphere, 2000 feet above sea level, sparsely settled community, nearest neighbor several miles away, and rarely ever seing other children. It was hard to realize diphtheria, but there could be no doubt about the diagnosis.

I at once began the administration of tinct. ferri chloride, quinine and chlorate potassa. Also tried local applications, which were, however, abandoned, after the second attempt, as the child seemed so frightened and struggled so hard, I was convinced the exhaustion following their use would more than counterbalance the good.

December 24, 8 a. m.—Child has less fever; pulse 155, weaker; respiration rapid and difficult; pseudo membrane has extended, covering the surfase of the pharynx, tonsils, and uvula, except a small strip in the centre. Have given regularly every two hours tinct. ferri chloride, gtt. 5; quinine, gr. 1; chlorate potassa gr. 5, and forced the child to take nourishment with stimulants every two hours. A strip of the membrane was separated by coughing and was found to be very firm and tenacious. Bowels have not acted for twenty hours; gave calomel.

8 p. m.—Condition remains about the same, except more force in circulation as result of nourishment and stimulants. Thorough evacuation of bowels from calomel.

December 25, 8 a. m.—No decided change in condition. 8 p. m. less fever; takes nourishment with less difficulty, but no improvement in throat.

December 26, 8 a. m.—Free from fever, pulse stronger, pseudo membrane has to some extent separated, and spots of mucous membrane can be seen on tonsils and pharynx. Discontinued tinct. ferri chloride and chlorate potassa, but kept up quinine. My object from the first was to saturate the child's system with the iron and potassa, which I think I have succeeded in doing.

The child steadily improved, and I did not see the case again, as parents lived thirty miles in the country.

Six weeks later the child was brought to me, the parents stating that for the past ten days they noticed she had difficulty in swallowing, and after lying in one position for some time she was unable to get up and turn over.

There was partial paralysis of the muscles of deglutition, neck, and lower extremities. Child walked with great difficulty, and her head fell forward, and chin rested upon her breast. There was also marked strabismus of both eyes.

I placed her upon iron, strychnine and quinine, and her recovery was steady. Three months later no trace of the paralysis was left, and at this writing, three months later, she has regained her usual flesh and is as robust as previous to attack of diphtheria.

It is an interesting question, how diphtheria causes paralysis, which does not come on in many cases for weeks after the attack. It must be from the direct effect of the diphtheric poison upon the nerves, as the paralysis is first manifested in the parts which were the seat of the exudation.

